# Identifying Genes Associated With Proliferation, Immunity and Thrombosis in Paroxysmal Nocturnal Haemoglobinuria

**DOI:** 10.1111/jcmm.70295

**Published:** 2024-12-13

**Authors:** Yali Du, David Wang, Qinglin Hu, Zhizhen Lai, Chen Yang, Huiwen He, Shuqing Wang, Huijuan Zhang, Peng Chen, Zepeng Li, Miao Chen, Bing Han

**Affiliations:** ^1^ Department of Hematology Peking Union Medical College Hospital, Chinese Academy of Medical Sciences and Peking Union Medical College Beijing China; ^2^ Medical Zhongcheng Limited Company Hangzhou China; ^3^ Department of Biophysics and Structural Biology Institute of Basic Medical Sciences, Chinese Academy of Medical Sciences & School of Basic Medicine, Peking Union Medical College Beijing China; ^4^ Department of Hematology, the Seventh Affiliated Hospital Sun Yat‐Sen University Guangzhou China

**Keywords:** clinical features, paroxysmal nocturnal haemoglobinuria, protein expression, whole‐exon sequencing, whole‐transcriptome sequencing

## Abstract

*PIGA* mutation cannot fully explain the proliferative advantage of abnormal clones and thrombosis tendency in paroxysmal nocturnal haemoglobinuria (PNH), and additional genes may play a role, justifying further investigation. CD59+ and CD59‐ peripheral blood mononuclear cells from six PNH patients were sorted and subjected to whole‐exon sequencing (WES) and whole‐transcriptome sequencing respectively. Six age‐ and sex‐matched healthy volunteers were enrolled as controls. Genes related to proliferation, immunity and thrombosis were selected by gene ontology (GO) analysis. The selected gene mutant alleles were then identified in the WES results for 40 patients and verified by the Sanger method in another 40 PNH patients. CD59+ and CD59‐ peripheral blood mononuclear cells from seven patients were sorted, and the RNA and protein expression levels of target genes were assessed via quantitative real‐time PCR (RT‐qPCR), flow cytometry and western blotting. The final selected genes were then related to clinical features to analyse. T‐cell activation–related genes were upregulated, whereas platelet degranulation, coagulation, haemostasis, leukocyte proliferation and platelet activation–related genes were downregulated in both CD59+ and CD59‐ cells. The mRNA or protein expression levels of SELP, FLT1, NRP1 and vWF were either different from those in healthy controls or different between CD59+ and CD59‐ cells. Moreover, platelet aggregation was greater in patients with mutations in these genes than in patients without such mutations. Except for *PIGA*, other genes may be involved in the proliferation and coagulopathy that occur in PNH patients.

## 1 Introduction

The proliferation advantage of PNH clones and tendency to promote thrombosis has always been a principal topic in paroxysmal nocturnal haemoglobinuria (PNH) research. Studies have suggested that mutations in *PIGA* lead cells harbouring PNH clones to escape T‐cell‐mediated autoimmune attack, resulting in resistance to apoptosis and subsequent proliferation [1]. However, approximately 42%–90% of the *PIGA* mutations reported in PNH patients are inconsistently identified by different sequencing methods [2, 3, 4]. Moreover, it was predicted that 1 in 10,000 cells would be expected to produce an HSC (haematopoietic stem cell) with a *PIG‐A* mutation in approximately 10% of healthy controls [5]. The frequency of numerous myeloid tumour‐related gene mutations is very low and decreases with the expansion of PNH clones [3]. Furthermore, large PNH clones are associated with thrombosis, but patients with small PNH clones can also develop thrombosis in some cases [4]. Therefore, *PIGA* mutation cannot fully explain the observed incidence of thrombosis in PNH patients, and the PNH clone itself does not have an inherent growth advantage [6]. Secondary mutations may increase the proliferation advantage of *PIGA* clones [7, 8, 9, 10], which justifies further investigation.

## 2 Methods

### 2.1 Patient Selection

**FIGURE 1:**
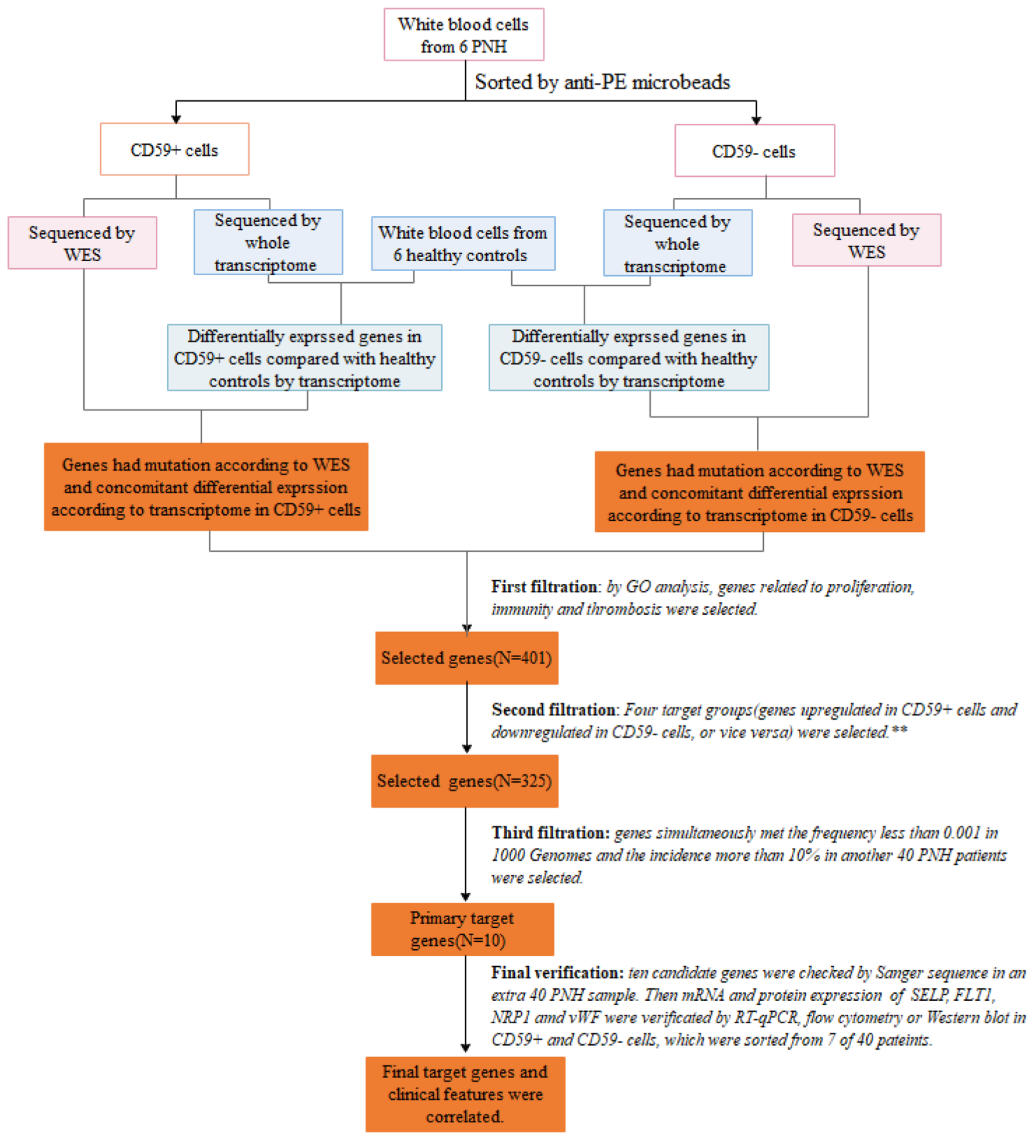
Flow chart of the entire study. Peripheral blood mononuclear cells from six patients with PNH and from six age‐ and sex‐matched healthy volunteers were sorted into CD59+ and CD59‐ cells and subjected to whole‐exon sequencing (WES) and whole‐transcriptome analysis respectively. Through whole‐transcriptome analysis, upregulated and downregulated genes were identified; the process of gene screening is described in detail in Figures S1 and S2. Pairwise comparisons of CD59+ versus CD59‐ in each individual patient were performed. Genes related to proliferation, apoptosis, immunity and thrombosis were selected by gene ontology (GO) analysis. Mutant alleles of the selected genes were subsequently identified via WES in another 40 PNH patients and verified by Sanger sequencing in this second 40‐patient cohort (primary target genes were selected). CD59+ and CD59‐ leukocytes from seven patients were sorted, and the RNA and protein expression levels of the target genes were verified via qPCR, flow cytometry and western blotting. The final selected target genes were tested for correlations with clinical features across all included patients. **Four target groups finally determined are shown in Figure S2. First screened target genes in detail are shown in Data S2 and clinical characteristics of different cohorts are listed in Table S3.

Patients with PNH newly diagnosed at Peking Union Medical College Hospital (PUMCH) between April 2019 and January 2022 according to previously established criteria [11] were included in this study. The patients were further stratified into classical PNH or AA (aplastic anaemia)/PNH subgroups according to the criteria established by the International PNH Interest Group. Briefly, patients who were diagnosed with PNH and had concomitant AA were categorised into the AA/PNH subgroup. The clinical information and laboratory results of the patients were recorded. Flow cytometry with fluorescent aerolysin (FLAER) was used to determine PNH clones, and the PNH clone size was calculated on the basis of the proportion of FLAER‐negative granulocytes, as detected by flow cytometry. All clinical data and blood samples were collected after written informed consent was signed by the patient. The clinical course and experimental protocol were in accordance with the Declaration of Helsinki and approved by the Ethics Committee of PUMCH. The experimental design is shown in Figure 1.

### 2.2 Cell Sorting of CD59‐ and CD59+ Cells via Microbeads

Peripheral blood mononuclear cells from six PNH patients and six age‐ and sex‐matched healthy controls were separated from peripheral blood samples via red blood cell lysis buffer (Lan Jie Bio, China). CD59‐ and CD59+ cells in the samples from the six patients were sorted as described previously [3]. Briefly, the cells were sorted via anti‐CD59 6PE (BD PharMingen, USA), anti‐PE microbeads (Miltenyi Biotec, Germany) and an immunomagnetic separation column (Miltenyi Biotec) according to the manufacturer's instructions. The purities of the CD59‐ and CD59+ fractions were confirmed by flow cytometry (BD Biosciences) and ranged from 94% to 98%. After sorting, the CD59+/− cells and control cells were subjected to WES or immediately cryopreserved in TRIzol (10^6^~10^7^ cells in 1 mL) for whole‐transcriptome sequencing.

### 2.3 Whole‐Exome Sequencing

DNA was extracted from the sorted cells of the above six patients, healthy control cells and cells from another 40 PNH patients via a PureLink Genomic DNA Mini Kit (Invitrogen) according to the manufacturer's instructions. Agarose gel electrophoresis was employed to assess the concentration, purity and integrity of the extracted DNA. WES was performed as follows: library construction was carried out via the Agilent SureSelect Human All Exon V6 Kit; the quality of the library was confirmed via a Qubit 2.0, NGS3K/Calliper and qPCR; and high‐throughput sequencing was conducted via an Illumina HiSeq2500 instrument (Illumina Inc.). Bioinformatic analysis was performed via the Genome Reference Consortium Human Genome Build 37 (hg19/GRCh37; ftp://ftp.ncbi.nlm.nih.gov/genomes/H_sapiens) as the reference genome. The average depth of sequencing was 127 × .

### 2.4 Whole‐Transcriptome Sequencing

Whole‐transcriptome sequencing was also performed on the sorted cells of the six patients via Novogene Source and sequenced via the Illumina high‐throughput sequencing platform. The average transcriptome sequencing data size was 6 Gb/sample. Bioinformatic analysis was conducted via the Genome Reference Consortium Human Genome Build 37 (hg19/GRCh37; ftp://ftp.ncbi.nlm.nih.gov/genomes/H_sapiens) as the reference genome.

### 2.5 Analysis of WES and Whole‐Transcriptome Sequencing Data

Genes were selected if they had a mutation according to WES and concomitant abnormal expression according to whole‐transcriptome sequencing of CD59+ and CD59‐ cells from the six patients. Compared with those in healthy controls, genes upregulated or downregulated in CD59+ and CD59‐ cells only or in combination or that differed between CD59+ and CD59‐ cells were selected. *R. 4.1.1* was used to perform gene ontology (GO) analysis by loading lists of significantly differentially expressed genes in CD59+ and CD59‐ cells separately. Genes enriched in functions related to proliferation, immunity and thrombosis were selected. The selected gene mutations were then screened from the WES results for the other 40 PNH patients, and mutations with a frequency of less than 0.1% in the 1000 Genomes database but greater than 10% in the PNH database were considered primary target genes. Interactions between primary target genes were analysed via the STRING (functional protein association network) (http://string‐db.org). The sequencing data were analysed with a Navicat Premium.

### 2.6 Sanger Sequencing

DNA was extracted from peripheral blood mononuclear cell samples from another 40 PNH patients as previously described. The primary target genes selected from the above procedures were further examined in these 40 patients via Sanger sequencing to identify the final target genes. Briefly, PCR amplification was performed according to the PCR touchdown amplification procedure (primers used for PCR and amplification were listed in Table S1 and Data S1). The products were purified, mixed with HIDI (highly deionised formamide) and sequenced at 3730 × L.

### 2.7 Cell Sorting for CD59‐ and CD59+ by Flow Cytometry and Detection of Target Proteins by Flow Cytometry

Gene mutations selected after the above procedures were further identified in terms of protein expression. Peripheral blood mononuclear cell samples from another seven patients who were followed from the second 40‐patient cohort were sorted by flow cytometry using anti‐CD59‐FITC and anti‐CD45‐BV421 antibodies. After sorting, CD59+/− cells were cryopreserved directly for western blotting and protein mass spectrometry (MALDI TOF MS, Bruker Daltonics, Billerica, MA) for protein expression assessment or cryopreserved in TRIzol (10^6^~10^7^ cells in 1 mL) for RNA expression assessment. Anti‐CD304‐PE (NRP1), anti‐FLT1‐APC (FLT1) and anti‐P selectin‐PE (SELP) were used for surface staining for target protein detection via flow cytometry. Blood samples from three healthy volunteers were taken as controls.

### 2.8 Quantitative Real‐Time qPCR

mRNA expression of the selected genes was also assessed in the above seven patients via quantitative real‐time RT‐PCR (RT‐qPCR), which was performed via SYBR Green I qPCR (Sangon Biotech) with appropriate primers (Table S2) and analysed via a LightCycler 480 II (Roche, Rotkreuz, Switzerland). All PCRs were performed in triplicate on 384‐well plates, and mRNA expression relative to that of the control β‐actin was calculated via the 2^−(∆∆Ct)^ method (primers for qPCR are listed in Table S2).

### 2.9 Western Blotting and Mass Spectrometry

Anti‐vWF was used for the detection of the target protein vWF via western blotting (WB), and β‐actin was used as an internal reference. All mAbs were purchased from commercial companies. The WB results were processed by ImageJ to produce quantitative data for further analysis.

The results of protein mass spectrometry were analysed via Mascott tools (http://www.matrixscience.com/ and https://web.ExPASy.org/peptide_mass/) for library identification and via https://www.uniprot.org/database for functional annotation.

### 2.10 Correlations of the Final Selected Genes With Clinical Features

The identified target genes were analysed with respect to clinical features such as PNH clone size, PNH subtype, response to immunosuppressive therapy (IST) for AA/PNH patients and thrombotic events.

### 2.11 Statistical Analysis

All the statistical analyses were performed via SPSS 26 and GraphPad PRISM version 8.0. Student's t‐test was used to determine the statistical significance of differences between two groups. Categorical variables were presented as frequencies and percentages. Group differences were determined by the chi‐square test or Fisher's exact test. A *p*‐value less than 0.05 was considered to indicate statistical significance.

## 3 Results

Here, peripheral mononuclear blood cells from six patients were sorted into CD59+ and CD59‐ cells and subjected to both whole‐exon sequencing (WES) and whole‐transcriptome sequencing (Figure 1) (Figures S1 and S2). Genes that may contribute to proliferation, immunity or thrombosis were identified. T‐cell activation–related genes were upregulated, whereas platelet degranulation, coagulation, haemostasis, leukocyte proliferation and platelet activation–related genes were downregulated in PNH patients compared with healthy controls (Figure S3A,B). Additionally, genes involved in neutrophil activation involved in the immune response, neutrophil activation, neutrophil degranulation and neutrophil‐mediated immunity were downregulated in CD59+ cells but upregulated in CD59‐ cells (Figure S3C,D). This finding is consistent with previous results [8] confirming that PNH patients have more activated immune attack and impaired haematopoiesis than healthy controls do [7, 10]. Compared with those in healthy controls, proliferation‐, coagulation‐ and haemostasis‐related genes were not only abnormally expressed in PNH patients but also differentially expressed between CD59+ and CD59‐ cells. The use of residual CD59+ cells had disadvantages, including limited proliferation and susceptibility to immune attack. Gene ontology (GO) analysis revealed differential regulation of 416 genes related to proliferation or apoptosis, 716 related to immunity and 141 related to thrombosis in PNH patients compared with healthy controls. We also evaluated genes upregulated in CD59+ cells and downregulated in CD59‐ cells or *vice versa* (Figures S2 and S3). As some genes were involved in multiple pathways, duplicate genes were removed, and a final set of 325 genes was selected for further analysis (Data S2).

The data for these 325 genes within the WES results from 40 patients were examined, and those with a mutation incidence greater than 10% in PNH but less than 0.001 in the 1000 Genomes Project were considered candidate genes. Finally, CARD11, vWF, MUC5B, ABCA13, FLT1, NRP1, SELP, SWAP70, EPHB2 and SLC15A4 were selected. Ten candidate genes were further characterised via Sanger sequencing in another 40 PNH patients. Eight genes were ultimately identified: NRP1 (10.0%, *N* = 4), SELP (12.5%, *N* = 5), vWF (2.5%, *N* = 1), SLC15A4 (5%, *N* = 2), FLT1 (2.5%, *N* = 1) and ABCA13 (2.5%, *N* = 1) (Figure S4). Information on the gene mutations detected by Sanger sequencing is presented in Table S6. Among them, mutations in ABCA13, FLT1, NRP1, SWAP70 and SLC15A4 are strongly associated with immunity or proliferation, whereas mutations in VWF, SELP and EPHB2 are associated mainly with thrombosis. However, genes may have multiple functions and interact with other genes. For example, SWAP70 mediates v‐GPCR‐induced endothelial cell plasticity and participates in NF‐κB signal transduction [12, 13], which is involved in apoptosis. Previous studies have indicated that ABCA13 amplification increases the risk of lymph node metastasis and is associated with poor outcomes [14]. SLC15A4 has been reported in studies of systemic lupus erythematosus (SLE) [15], which is a typical autoimmune disease.

Ten candidate genes were analysed via STRING protein interaction network analysis. SELP, FLT1, NRP1 and vWF were significantly connected with each other (Figure S4A). The mRNA or protein expression of these four genes was subsequently measured via qPCR and flow cytometry (or western blotting).

**FIGURE 2:**
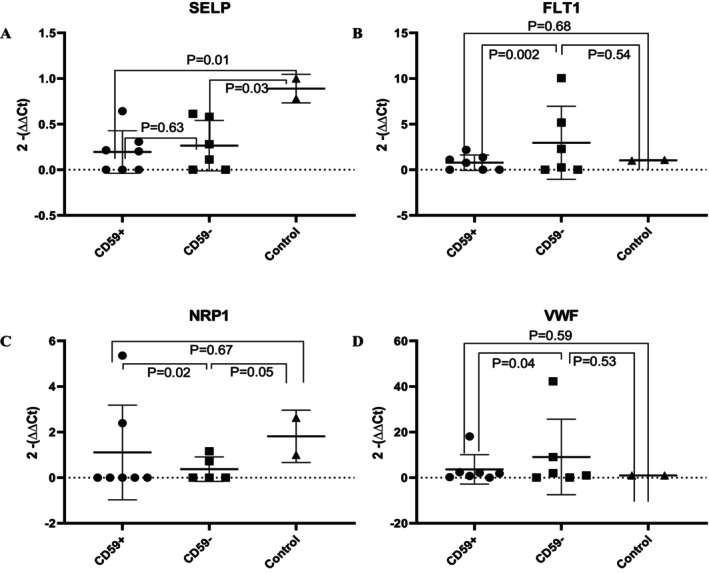
Gene expression of candidate genes via RT‐qPCR analysis. The expression levels of four selected candidate genes were evaluated in sorted CD59+ and CD59‐ cells from seven PNH patients and three healthy controls. Relative RNA expression was calculated via normalisation to β‐Actin expression and is shown as the fold change relative to healthy controls. (A) SELP gene; (B) FLT1 gene; (C) NRP1 gene; (D) vWF gene. The mRNA levels of these target genes in PNH patients were either different from those in healthy controls or different between CD59+ and CD59‐ cells. The expression of SELP was similar in CD59+ and CD59‐ cells (*p* = 0.63). However, SELP expression in CD59+ (*p* = 0.01) and CD59‐ (*p* = 0.03) cells was significantly lower in patients than in healthy controls. The expression of FLT1 and vWF on CD59+ cells was significantly lower than that on CD59‐ cells (*p* = 0.002 and 0.04 respectively) but was similar between CD59+ cells from PNH patients and healthy controls (*p* = 0.68 and 0.59 respectively). NRP1 expression was significantly greater on CD59+ cells than on CD59‐ cells (*p* = 0.02) but was similar between CD59+ cells from patients and healthy controls (*p* = 0.67).

**FIGURE 3:**
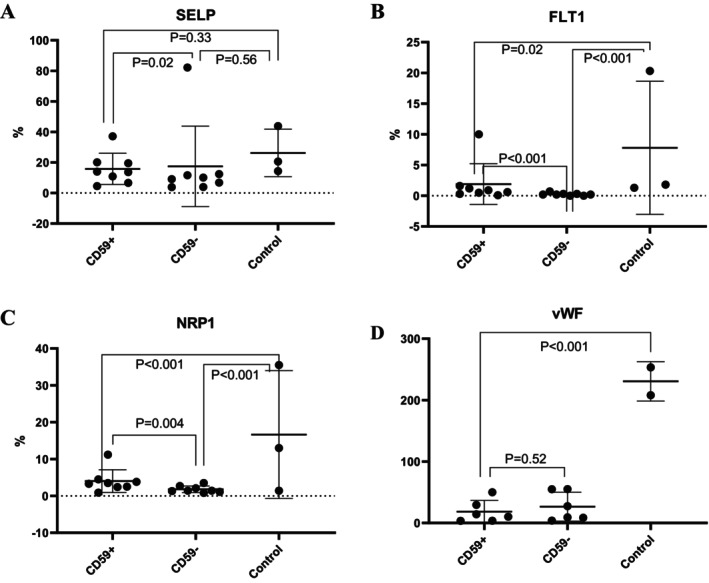
Validation of protein expression in CD59+ and CD59‐ cells from PNH patients (*n* = 7) and healthy controls (*n* = 3) by western blotting or flow cytometry. The expression of (A) SELP, (B) FLT1 and (C) NRP1 was analysed by flow cytometry. (D) The expression of vWF in CD59+ cells, CD59‐ cells and healthy control cells was analysed by western blotting. The protein levels of these target genes in PNH patients were either different from those in normal controls or different between CD59+ and CD59‐ cells. SELP expression was lower on CD59+ cells than on CD59‐ cells (*p* = 0.02) but was similar between CD59+ cells from patients and healthy controls (*p* = 0.33). FLT1 expression was greater on CD59+ cells than on CD59‐ cells (*p* < 0.001) but lower on CD59+ cells from patients than on cells from healthy controls (*p* = 0.02). In patients, NRP1 expression was greater on CD59+ cells than on CD59‐ cells (*p* = 0.004) but lower on CD59+ cells from patients than on those from healthy controls (*p* < 0.001). The vWF expression levels detected by Western blotting were similar between CD59+ and CD59‐ cells (*p* = 0.52) but were lower in cells from patients than in those from healthy controls (*p* < 0.001).

The mRNA expression levels of SELP, FLT1, NRP1 and vWF were verified via qPCR. The expression of SELP was similar in CD59+ and CD59‐ cells (*p* = 0.63). However, SELP expression in CD59+ (*p* = 0.01) and CD59‐ (*p* = 0.03) cells was significantly lower in patients than in healthy controls. The expression of FLT1 and vWF on CD59+ cells was significantly lower than that on CD59‐ cells (*p* = 0.002 and 0.04 respectively) but was similar between CD59+ cells from PNH patients and healthy controls (*p* = 0.68 and 0.59 respectively). NRP1 expression was significantly greater on CD59+ cells than on CD59‐ cells (*p* = 0.02) but was similar between CD59+ cells from patients and healthy controls (*p* = 0.67) (Figure 2A–D). The protein expression of SELP, FLT1 and NRP1 was detected via flow cytometry. SELP expression was lower on CD59+ cells than on CD59‐ cells (*p* = 0.02) but was similar between CD59+ cells from patients and healthy controls (*p* = 0.33). FLT1 expression was greater on CD59+ cells than on CD59‐ cells (*p* < 0.001) but lower on CD59+ cells from patients than on cells from healthy controls (*p* = 0.02). In patients, NRP1 expression was greater on CD59+ cells than on CD59‐ cells (*p* = 0.004) but lower on CD59+ cells from patients than on those from healthy controls (*p* < 0.001). The vWF expression levels detected by Western blotting were similar between CD59+ and CD59‐ cells (*p* = 0.52) but were lower in cells from patients than in those from healthy controls (*p* < 0.001) (Figure 3A–D).

Coomassie brilliant blue staining of proteins separated by SDS‐PAGE under the same loading conditions revealed significant differences in protein expression between healthy controls and PNH patients (Figure S5 and Table S5). The bands were sent for mass spectrometry detection, and the results suggested that CD59+ cells expressed antiproliferative proteins and that CD59‐ cells expressed proteins related to regulating cell growth and proliferation (Table S5).

Finally, the correlations between mutations in the 10 candidate genes and the clinical features of PNH patients were analysed. A greater proportion of patients with small PNH clones (≤ 10%) had SWAP70, SLC15A4 and ABCA13 mutations (*p* < 0.05). In addition, we found that a greater proportion of patients with thrombosis than patients without thrombosis carried EpHB2 mutations (*p* = 0.015), SWAP70 mutations (*p* = 0.039), ABCA13 mutations (*p* = 0.01) and SLC15A4 mutations (*p* = 0.004). ADP‐induced platelet aggregation (61.33% ± 8.22% vs. 52.75% ± 18.54%, *p* = 0.02), collagen‐induced platelet aggregation (68.3% ± 4.08% vs. 57.39% ± 17.96%, *p* < 0.001) and ristomycin‐induced platelet aggregation (66.2% ± 8.04% vs. 56.57% ± 19.22%, *p* = 0.009) were greater in patients with mutations in these candidate genes than in patients without such mutations.

## 4 Discussion

The research object of this study was peripheral blood mononuclear cells, including lymphocytes, granulocytes and monocytes, which were analysed via WES, transcriptome sequencing and subsequent qPCR. Previous studies have performed directed panel sequencing of stem cell and lymphocyte subsets (T cells) [7], but sequencing of peripheral blood mononuclear cells in PNH patients has not been reported. In our pilot experiments, we found that a peripheral blood sample of approximately 20 mL was required to obtain sufficient cells for PCR and transcriptome analysis (due to the different peripheral blood white blood cell counts in each patient). Moreover, the time and cost of cell sorting have doubled. Therefore, peripheral blood mononuclear cells, which have not been used previously, were ultimately identified as research objects. The controls were cells from healthy individuals rather than from the fingernails or saliva of patients. The transcriptomes of PNH patients and healthy controls were sequenced to identify differentially expressed genes, and the six PNH samples were subjected to WES simultaneously. The required sample size of 6 was estimated by a professional sequencing team. A pairwise comparison of CD59+ versus CD59‐ cells in each individual patient was performed. None of the patients received component inhibitors.

We found that T‐cell activation–related genes were upregulated in PNH patients compared with healthy controls but that platelet degranulation, coagulation, haemostasis, leukocyte proliferation and platelet activation–related genes were downregulated. Leukocyte proliferation, apoptosis regulation, immune activation, platelet activation and coagulation regulation genes were also downregulated in CD59+ cells compared with those in CD59‐ cells. This finding is similar to previous results [8, 16, 17, 18, 19]. These findings confirm that PNH patients have more activated immune attack and impaired haematopoiesis than healthy controls do. Proliferation‐, coagulation‐ and haemostasis‐related genes were not only abnormally expressed but also expressed unevenly on CD59+ and CD59‐ cells. Residual CD59+ cells have the disadvantage of proliferation and susceptibility to immune attack, which is consistent with previous studies [10, 18, 20]. Eculizumab reduced the incidence of thrombosis in PNH patients [21], which proves that excessive activation of the complement system is related to thrombosis. Previous research reported the mechanism of thrombosis included platelet activation, toxicity of free haemoglobin, nitric oxide depletion, absence of other glycosylphosphatidylinositol‐linked proteins such as urokinase‐type plasminogen activator receptor and endothelial dysfunction [22]. The number of extracellular vesicles released from the plasma of PNH patients increases, and there are differences in metabolism in plasma exosomes, thus pointing out prognostic biomarkers for the risk of thrombosis in PNH patients [23]. Since the formation of thrombosis requires the participation of coagulation factors and platelet activation. It was found that platelet degranulation, coagulation, haemostasis, leukocyte proliferation and platelet activation–related genes in PNH patients are downregulated by comparing with normal people in this article. Therefore, it implied that PNH patients had abnormal blood coagulation and bleeding mechanisms compared with normal people, and were thus prone to thrombosis.

A greater proportion of patients with small PNH clones (≤ 10%) had SWAP70, SLC15A4 and ABCA13 mutations (*p* < 0.05). This can be explained by the fact that these patients may have greater immune‐related pathogenesis and thus a greater frequency of immune‐related gene mutations. In previous reports, the IST mentioned in the article is mainly used for BMF/PNH patients [24]. There are 38 patients who received IST included in this analysis. There are 24 cases in the effective IST group and 14 cases in the ineffective group (specific information is shown in Table S4). However, the response to IST in BMF/PNH patients did not correlate with any of these genes (*p* > 0.99). Overall, patients with BMF/PNH were generally sensitive to IST, and high expression of these immune‐related genes is a reflection of disease characteristics rather than a predictor of treatment response.

Previous in vitro studies have shown that an imbalance in the EPHB2–EPHB4 ratio in children with cerebral arteriovenous malformation is related to endothelial cell dysfunction [25]. SLC15A4 is preferentially expressed in immune cells and plays critical roles in the pathogenesis of lupus in murine models [15]. On the other hand, SLE, like PNH, is characterised by complement activation and thrombogenesis, which can explain, to some extent, our finding that patients with thrombosis are likely to have higher SLC15A4 or ABCA13 mutation incidence than patients without thrombosis.

The mRNA or protein expression levels of SELP, FLT1, NRP1 and vWF were either different from those in healthy controls or different between CD59+ and CD59‐ cells. Moreover, platelet aggregation was greater in patients with mutations in target genes than in patients without such mutations. The results from mass spectrometry detection also revealed that antiproliferative proteins expressed in CD59+ cells and proteins related to regulating cell growth and proliferation were expressed in CD59‐ cells. Overall, our analyses indicated that T‐cell activation–related genes were upregulated in PNH patients than in healthy, leukocyte proliferation, apoptosis regulation, and immune activation–related genes were downregulated in CD59+ cells than in CD59‐ cells. Patients with mutations in candidate genes had higher thrombosis portion and different platelet aggregation than patients without such mutations. Therefore, we inferred that additional genes may contribute to the pathogenesis of thrombosis tendency or clone proliferation in PNH.

Variations in the expression of proliferation‐associated and thrombosis‐associated genes and proteins were detected among individuals with CD59+ or CD59‐ disease and among healthy controls, suggesting the need for further detailed functional analysis of genes other than *PIGA* in the pathogenesis of PNH. The advantage of this study is that it enables a narrowing of target gene screening from various perspectives. Kohei Hosokawa et al. reported increased expression of the TNFAIP3 gene and protein in T cells at PNH [7], and we also reported that TNFAIP3 was more highly expressed in CD59+ and CD59‐ cells in patients than in healthy individuals, indicating that this analysis is reliable. However, cell functions do not necessarily change on the basis of differences in DNA sequence or transcript or protein levels. Therefore, functional genes may be missed when focusing on the DNA–transcript–protein axis. Subsequent research should use mass spectrometry to investigate differences in proteins in larger cohorts of PNH patients and healthy controls.

## Author Contributions

**Yali Du:** conceptualization (lead), data curation (lead), formal analysis (lead), investigation (lead), methodology (lead), visualization (lead), writing – original draft (lead), writing – review and editing (lead). **David Wang:** software (lead). **Qinglin Hu:** formal analysis (supporting). **Zhizhen Lai:** methodology (equal). **Chen Yang:** investigation (supporting). **Huiwen He:** formal analysis (supporting), methodology (supporting). **Shuqing Wang:** formal analysis (supporting), methodology (supporting). **Huijuan Zhang:** methodology (supporting). **Peng Chen:** data curation (supporting), formal analysis (supporting), methodology (supporting), software (supporting). **Zepeng Li:** resources (supporting). **Miao Chen:** investigation (supporting), writing – review and editing (supporting). **Bing Han:** supervision (lead), writing – original draft (supporting), writing – review and editing (supporting).

## Conflicts of Interest

The authors declare no conflicts of interest.

## Supporting information


**FIGURE S1.** (A) Distribution of differential genes in the respective CD59+ set, CD59‐ set and CD59+/CD59‐ common intersection set from samples 1–6 respectively. (B) Summarisation of three data sets of samples 1–6. Elements in CD59+ set include ‘1, 2, 3, 4, 6’, elements in CD59‐ set include ‘4, 5, 6, 7, 8, 9, 10, 12’ and intersection elements of CD59+/CD59‐ are ‘3, 4, 5, 6, 11’. It was found that element ‘3’ exists in CD59+ set and CD59+/CD59‐ common intersection, elements ‘4,6’ exist in CD59+ set, CD59‐ set and CD59+/CD59‐ common intersection and element ‘5’ exists in CD59‐ set and CD59+/CD59‐ common intersection. After pooling of samples 1–6, there are still repeated elements among the three sets. Then, processed by ‘purification’, and another data set is generated with ‘Dirty’. Because Dirty sets are derived from CD59+ and/or CD59‐ sets, they are considered to represent unsorted PNH. Finally, four data sets of CD59+ specific, CD59‐ specific, CD59+ and CD59‐ intersection and Dirty are formed. (C) Upregulated genes were represented by elements ‘1,2,3,4,5,6,7,8,9,11,12’, while downregulated genes were illustrated by elements ‘11,12,13,14,15,16,17,18,19,111,112’. Through GO analysis of upregulated and downregulated differential genes in CD59‐ specific, CD59+ specific, CD59+ and CD59‐ intersection specific and Dirty, target genes were further narrowed into six groups (Marked by thick red checkmark in Figure C), including genes upregulated in CD59+ but downregulated CD59‐, upregulated in CD59‐ but downregulated CD59+, upregulated in CD59+/CD59‐ common intersection, downregulated in CD59+/CD59‐ common intersection, upregulated in Dirty and downregulated in Dirty. WES and transcriptome were analysed in combination. Genes were selected if they had a mutation according to WES and concomitant abnormal expression according to whole‐transcriptome sequencing of CD59+ and CD59‐ cells from the six patients.

**FIGURE S2.** (A) Through GO analysis of upregulated and downregulated differential genes in CD59+ specific, CD59‐ specific, CD59+ and CD59‐ intersection specific and Dirty, target genes of thrombosis, immunity, proliferation and apoptosis after GO analysis in each group were screened (number of target genes is shown in the figure). (B) Four target groups were final determined (background is circled in red in Figure B), including genes upregulated in CD59+ but downregulated CD59‐, genes upregulated in CD59‐ but downregulated CD59+, genes upregulated in Dirty and genes downregulated in Dirty (Gene names in detail are in Data S2).

**FIGURE S3.** Dirty sets are derived from CD59+ and/or CD59‐ sets, they are considered to represent unsorted PNH. (A) Upregulated differential genes were enriched to the top 10 in Dirty set included neutrophil activation, T‐cell activation, neutrophil degranulation, neutrophil activation involved in immune response, neutrophil mediated immunity, leukocyte cell–cell adhesion, positive regulation of cell adhesion, regulation of cell–cell adhesion, positive regulation of leukocyte activation, positive regulation of cell activation. Neutrophil activation and T‐cell activation–related were enriched by upregulated differential genes in PNH patients compared with the healthy controls (*p* < 0.05). (B) Downregulated differential genes were enriched to the top 10 in Dirty set including platelet degranulation, blood coagulation, haemostasis, coagulation, neutrophil activation, leukocyte proliferation, neutrophil degranulation, neutrophil activation involved in immune response, neutrophil‐mediated immunity. Platelet degranulation, blood coagulation, haemostasis and coagulation in PNH patients compared with healthy controls (*p* < 0.05). (C) Downregulated differential genes were enriched to the top in CD59+ set including neutrophil activation, neutrophil activation involved in immune response, neutrophil degranulation, neutrophil‐mediated immunity, positive regulation of cytokine production, regulation of cysteine‐type endopeptidase activity involved in apoptotic process, T‐cell activation, regulation of cysteine‐type endopeptidase activity, regulation of peptidase activity, regulation of endopeptidase activity. (D) Upregulated differential genes were enriched to the top 10 in CD59‐ set included neutrophil activation involved in immune response, neutrophil activation, neutrophil degranulation, neutrophil‐mediated immunity, type I interferon signalling pathway, cellular response to type I interferon, response to type I interferon, cellular response to chemical stress, positive regulation of defence response, defence response to virus.

**FIGURE S4.** (A) Gene correlation analysis via the STRING protein interaction network shows a significant correlation between SELP, vWF, FLT1 and NRP1. (B) Sanger sequencing results of selected genes. SELP (rs116959152), SELP (rs141287418), NRP1 (rs200381308), FLT1 (rs2296191), vWF (rs139196998), SLC15A4 (rs536892230), SLC15A4 (rs200862556) and ABCA13 (rs148629439) were identified (details are shown in Table S6).

**FIGURE S5.** (A) The expression of vWF in CD59+ cells, CD59‐ cells and healthy control cells was analysed by western blotting. (B) Band ‘a’ indicates a significant difference in protein expression between healthy controls and PNH patients by SDS–PAGE with Coomassie brilliant blue staining under the same loading conditions.

**TABLE S1.** Primers for Sanger sequencing.

**TABLE S2.** Primers for qPCR.

**TABLE S3.** Clinical characteristics of the different cohorts.

**TABLE S4.** Clinical characteristics of patients treated with immunosuppressive therapy (IST).

**TABLE S5.** Results of mass spectrometry.

TABLE S6.

Data S1.

Data S2.

## Data Availability

As required by the journal's data policy, this manuscript does not involve the generation of new datasets. Therefore, there is currently no data available for sharing. Any inquiries regarding this research can be directed to the corresponding author.
