# Supplementary material for: Identifying Genes Associated With Proliferation, Immunity and Thrombosis in Paroxysmal Nocturnal Haemoglobinuria
Source: J Cell Mol Med. 2024 Dec 13;28(23):e70295. doi: 10.1111/jcmm.70295 (PMC11640899; doi:10.1111/jcmm.70295)
Supplement: Supplementary file 1 — FIGURE S1. (A) Distribution of differential genes in the respective CD59+ set, CD59‐ set and CD59+/CD59‐ common intersection set from samples 1–6 respectively. (B) Summarisation of three data sets of samples 1–6. Elements in CD59+ set include ‘1, 2, 3, 4, 6’, elements in CD59‐ set include ‘4, 5, 6, 7, 8, 9, 10, 12’ and intersection elements of CD59+/CD59‐ are ‘3, 4, 5, 6, 11’. It was found that element ‘3’ exists in CD59+ set and CD59+/CD59‐ common intersection, elements ‘4,6’ exist in CD59+ set, CD59‐ set and CD59+/CD59‐ common intersection and element ‘5’ exists in CD59‐ set and CD59+/CD59‐ common intersection. After pooling of samples 1–6, there are still repeated elements among the three sets. Then, processed by ‘purification’, and another data set is generated with ‘Dirty’. Because Dirty sets are derived from CD59+ and/or CD59‐ sets, they are considered to represent unsorted PNH. Finally, four data sets of CD59+ specific, CD59‐ specific, CD59+ and CD59‐ intersection and Dirty are formed. (C) Upregulated genes were represented by elements ‘1,2,3,4,5,6,7,8,9,11,12’, while downregulated genes were illustrated by elements ‘11,12,13,14,15,16,17,18,19,111,112’. Through GO analysis of upregulated and downregulated differential genes in CD59‐ specific, CD59+ specific, CD59+ and CD59‐ intersection specific and Dirty, target genes were further narrowed into six groups (Marked by thick red checkmark in Figure C), including genes upregulated in CD59+ but downregulated CD59‐, upregulated in CD59‐ but downregulated CD59+, upregulated in CD59+/CD59‐ common intersection, downregulated in CD59+/CD59‐ common intersection, upregulated in Dirty and downregulated in Dirty. WES and transcriptome were analysed in combination. Genes were selected if they had a mutation according to WES and concomitant abnormal expression according to whole‐transcriptome sequencing of CD59+ and CD59‐ cells from the six patients. [file JCMM-28-e70295-s011.docx]

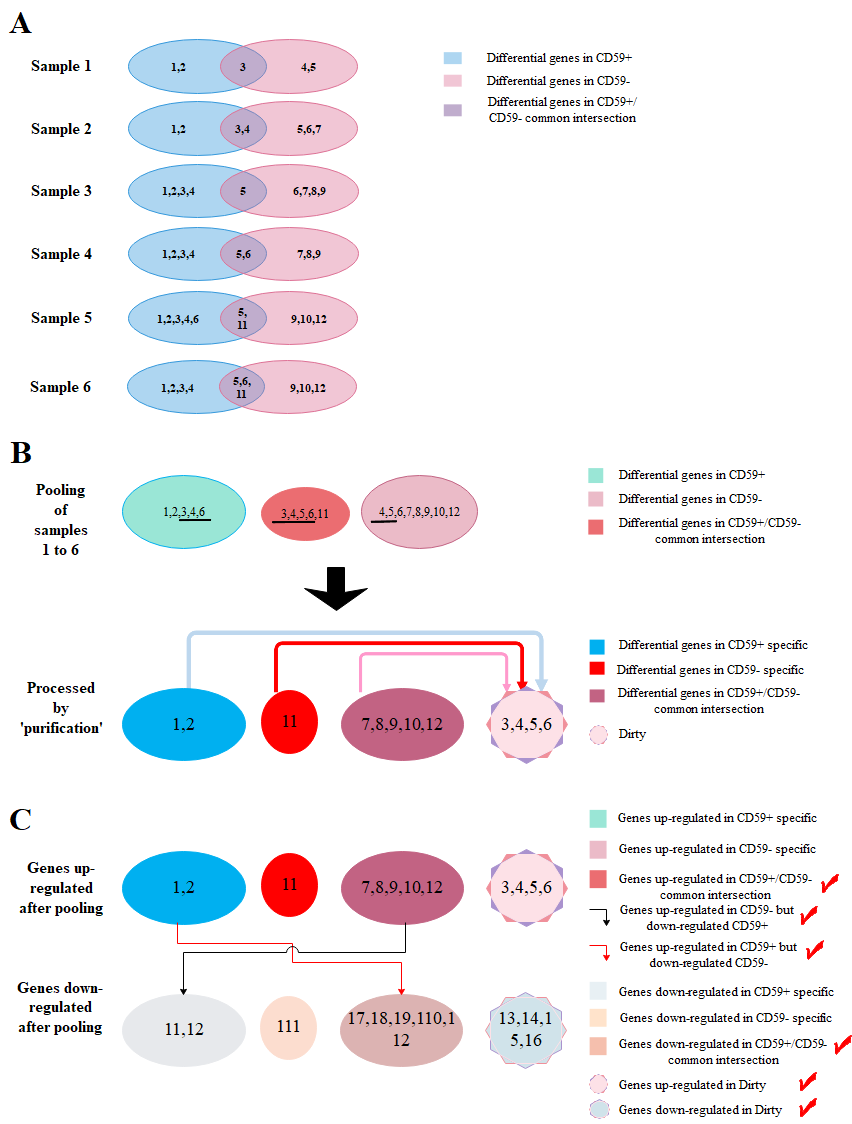


Supplementary Figure 1. A. Distribution of differential genes in the respective CD59+ set, CD59- set, and CD59+/CD59- common intersection set from samples 1-6 respectively. B. Summarize three data sets of samples 1 to 6. Elements in CD59+ set include ‘1, 2, 3, 4, 6’, elements in CD59- set include ‘4, 5, 6, 7, 8, 9, 10, 12’, and intersection elements of CD59+/CD59- are ‘3, 4, 5, 6, 11’. It was found that element ‘3’ exists in CD59+ set and CD59+/CD59- common intersection, element ‘4,6’ exist in CD59+ set, CD59- set and CD59+/CD59- common intersection, and element ‘5’ exists in CD59- set and CD59+/CD59- common intersection. After pooling of samples 1 to 6, there are still repeated elements among the three sets. Then processed by ‘**purification**’, and another data set is generated with ‘Dirty’. Because Dirty sets are derived from CD59+ and/or CD59- sets, they are considered to represent unsorted PNH. Finally, four data sets of CD59+ specific, CD59- specific, CD59+ and CD59- intersection and Dirty are formed. C. Upregulated genes were represented by elements ‘1,2,3,4,5,6,7,8,9,11,12’, while downregulated genes were illustrated by elements ‘11,12,13,14,15,16,17,18,19,111,112’. Through GO analysis of upregulated and downregulated differential genes in CD59- specific, CD59+ specific, CD59+ and CD59- intersection specific and Dirty, target genes were further narrowed in six groups(Marked by thick red checkmark in Figure C.), including genes up-regulated in CD59+ but down-regulated CD59-, up-regulated in CD59- but down-regulated CD59+, up-regulated in CD59+/CD59- common intersection, down-regulated in CD59+/CD59- common intersection, up-regulated in Dirty and down-regulated in Dirty. WES and transcriptome were analyzed in combination. Genes were selected if they had a mutation according to WES and concomitant abnormal expression according to whole-transcriptome sequencing of CD59+ and CD59- cells from the 6 patients.

The target genes of thrombosis, immunity, proliferation and apoptosis after GO analysis in each group were screened.
