# Supplementary material for: Identifying Genes Associated With Proliferation, Immunity and Thrombosis in Paroxysmal Nocturnal Haemoglobinuria
Source: J Cell Mol Med. 2024 Dec 13;28(23):e70295. doi: 10.1111/jcmm.70295 (PMC11640899; doi:10.1111/jcmm.70295)
Supplement: Supplementary file 2 — FIGURE S2. (A) Through GO analysis of upregulated and downregulated differential genes in CD59+ specific, CD59‐ specific, CD59+ and CD59‐ intersection specific and Dirty, target genes of thrombosis, immunity, proliferation and apoptosis after GO analysis in each group were screened (number of target genes is shown in the figure). (B) Four target groups were final determined (background is circled in red in Figure B), including genes upregulated in CD59+ but downregulated CD59‐, genes upregulated in CD59‐ but downregulated CD59+, genes upregulated in Dirty and genes downregulated in Dirty (Gene names in detail are in Data S2). [file JCMM-28-e70295-s003.docx]

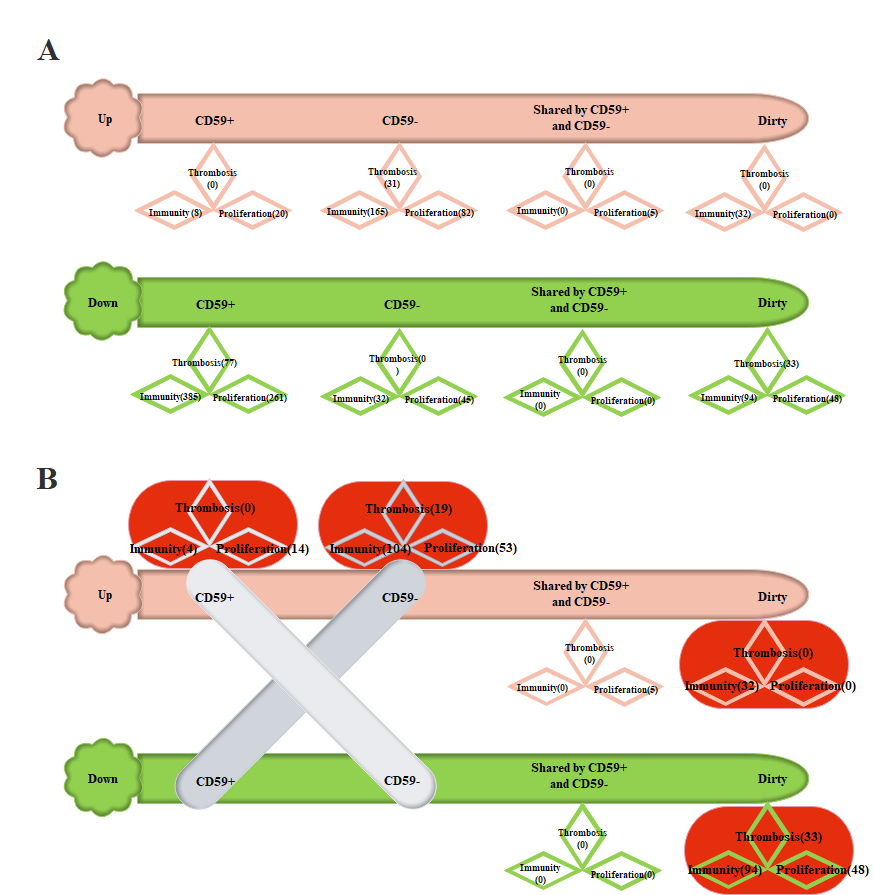


Supplementary Figure2. A. Through GO analysis of up-regulated and down-regulated differential genes in CD59+ specific, CD59- specific, CD59+ and CD59- intersection specific and Dirty, target genes of thrombosis, immunity, proliferation and apoptosis after GO analysis in each group were screened (Number of target genes was shown in the figure). B. Four target groups were final determined (Background were circled in red in figure B), including genes up-regulated in CD59+ but down-regulated CD59-, genes up-regulated in CD59- but down-regulated CD59+, genes up-regulated in Dirty and genes down-regulated in Dirty (Gene names in detail are in supplementary data 2.).
