# Supplementary material for: Identifying Genes Associated With Proliferation, Immunity and Thrombosis in Paroxysmal Nocturnal Haemoglobinuria
Source: J Cell Mol Med. 2024 Dec 13;28(23):e70295. doi: 10.1111/jcmm.70295 (PMC11640899; doi:10.1111/jcmm.70295)
Supplement: Supplementary file 3 — FIGURE S3. Dirty sets are derived from CD59+ and/or CD59‐ sets, they are considered to represent unsorted PNH. (A) Upregulated differential genes were enriched to the top 10 in Dirty set included neutrophil activation, T‐cell activation, neutrophil degranulation, neutrophil activation involved in immune response, neutrophil mediated immunity, leukocyte cell–cell adhesion, positive regulation of cell adhesion, regulation of cell–cell adhesion, positive regulation of leukocyte activation, positive regulation of cell activation. Neutrophil activation and T‐cell activation–related were enriched by upregulated differential genes in PNH patients compared with the healthy controls (p < 0.05). (B) Downregulated differential genes were enriched to the top 10 in Dirty set including platelet degranulation, blood coagulation, haemostasis, coagulation, neutrophil activation, leukocyte proliferation, neutrophil degranulation, neutrophil activation involved in immune response, neutrophil‐mediated immunity. Platelet degranulation, blood coagulation, haemostasis and coagulation in PNH patients compared with healthy controls (p < 0.05). (C) Downregulated differential genes were enriched to the top in CD59+ set including neutrophil activation, neutrophil activation involved in immune response, neutrophil degranulation, neutrophil‐mediated immunity, positive regulation of cytokine production, regulation of cysteine‐type endopeptidase activity involved in apoptotic process, T‐cell activation, regulation of cysteine‐type endopeptidase activity, regulation of peptidase activity, regulation of endopeptidase activity. (D) Upregulated differential genes were enriched to the top 10 in CD59‐ set included neutrophil activation involved in immune response, neutrophil activation, neutrophil degranulation, neutrophil‐mediated immunity, type I interferon signalling pathway, cellular response to type I interferon, response to type I interferon, cellular response to chemical stress, positive regulati [file JCMM-28-e70295-s006.docx]

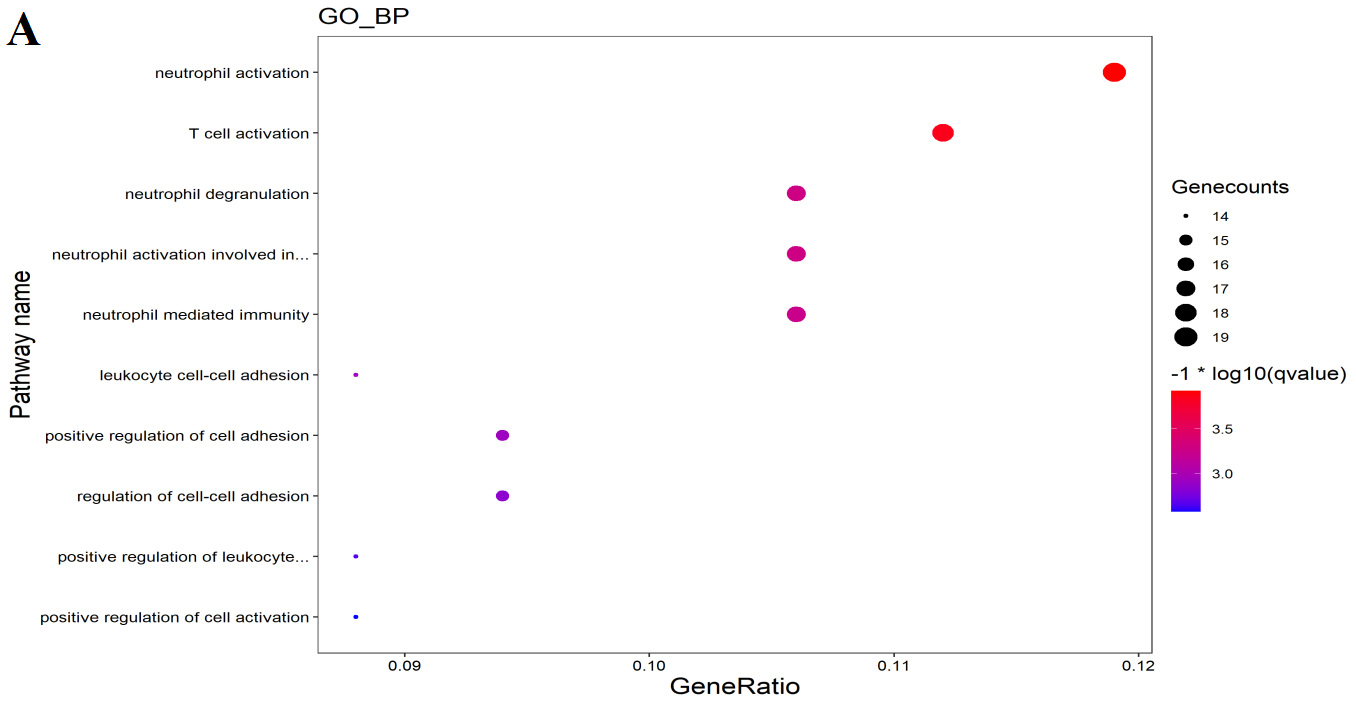

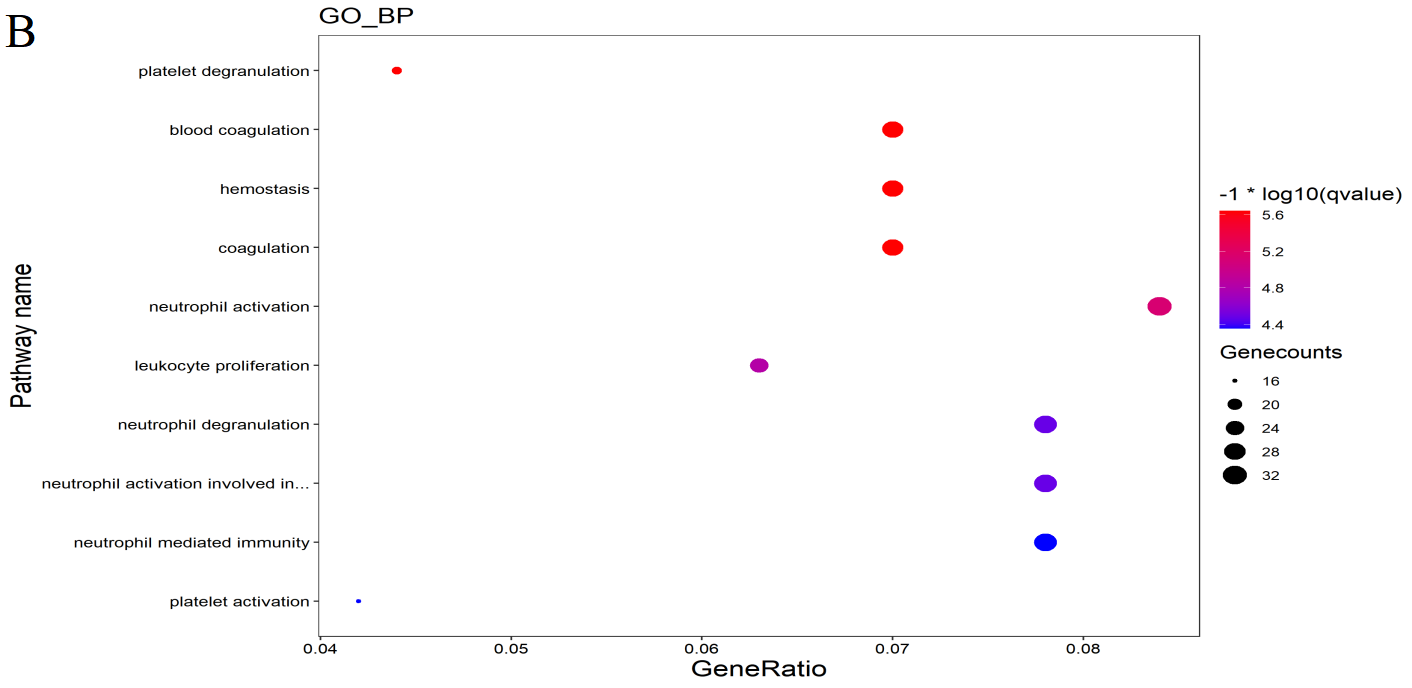


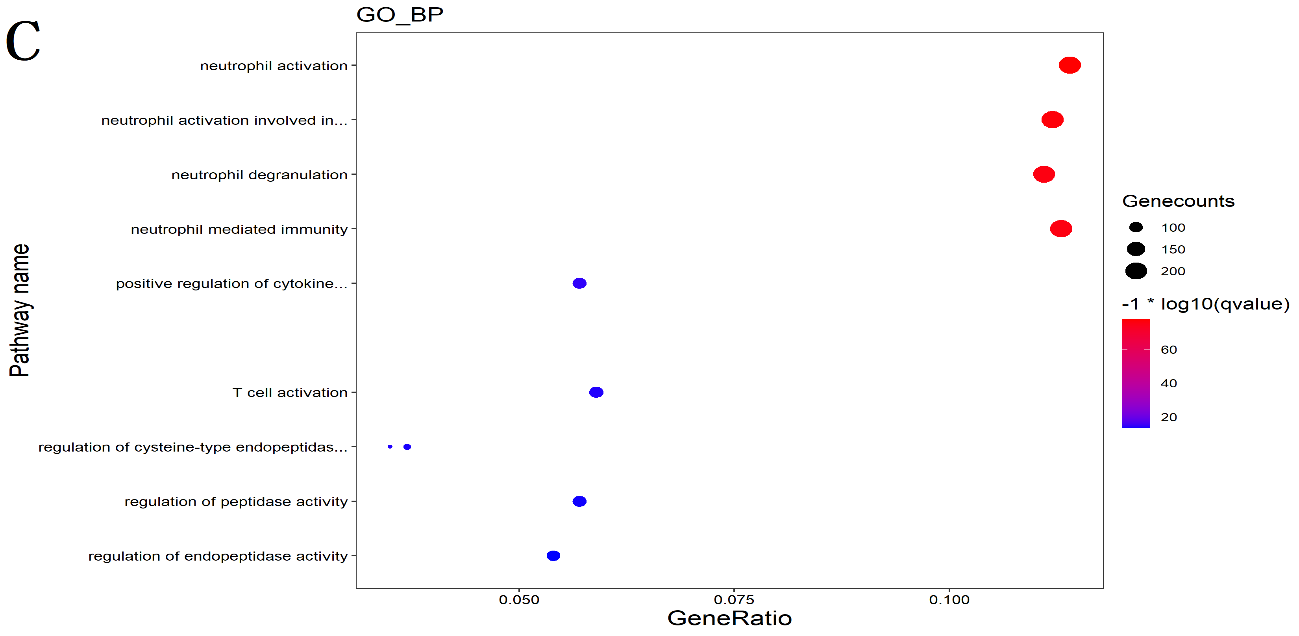


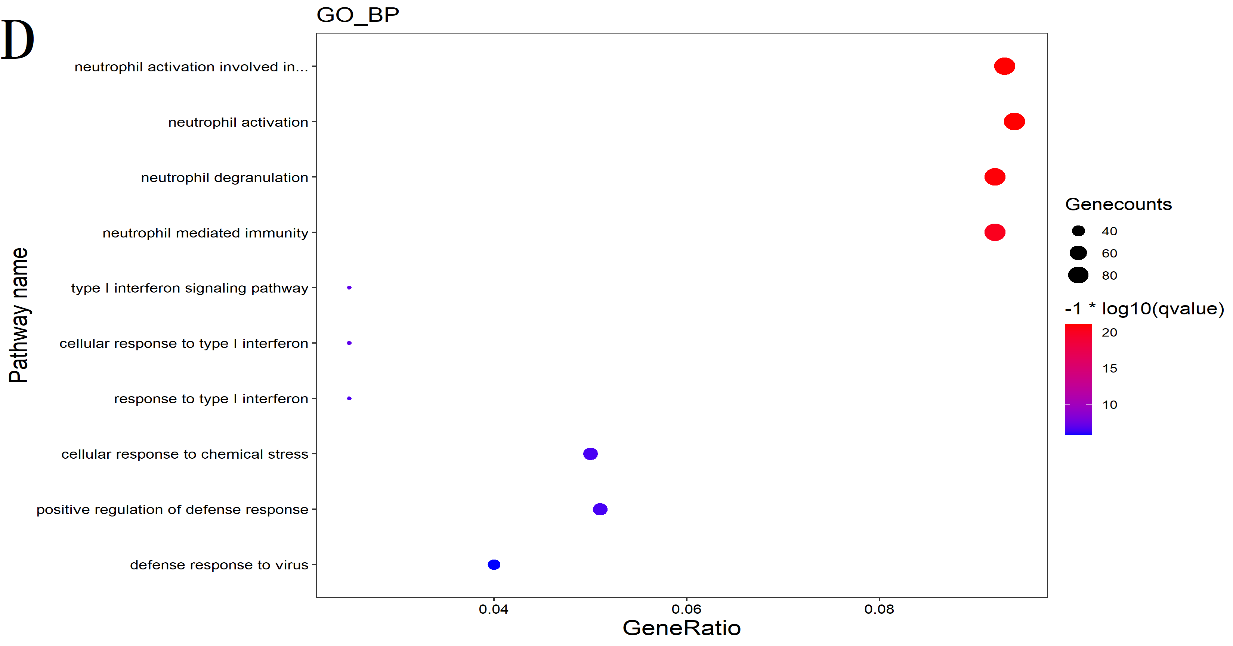


Supplementary Figure3.Dirty sets are derived from CD59+ and/or CD59- sets, they are considered to represent unsorted PNH. **A.** Upregulated differential genes were enriched to the top 10 in Dirty set included neutrophil activation, T cell activation, neutrophil degranulation, neutrophil activation involved in immune response, neutrophil mediated immunity, leukocyte cell-cell adhesion, positive regulation of cell adhesion, regulation of cell-cell adhesion, positive regulation of leukocyte activation, positive regulation of cell activation. Neutrophil activation and T cell activation-related were enriched by up-regulated differential genes in PNH patients compared with the healthy controls(P<0.05). **B.** Downregulated differential genes were enriched to the top 10 in Dirty set included platelet degranulation, blood coagulation, hemostasis, coagulation, neutrophil activation, leukocyte proliferation, neutrophil degranulation, neutrophil activation involved in immune response,neutrophil mediated immunity. Platelet degranulation, blood coagulation, hemostasis, and coagulation in PNH patients compared with healthy controls(P<0.05). **C.** Downregulated differential genes were enriched to the top in CD59+ set included neutrophil activation, neutrophil activation involved in immune response, neutrophil degranulation, neutrophil mediated immunity, positive regulation of cytokine production, regulation of cysteine-type endopeptidase activity involved in apoptotic process, T cell activation, regulation of cysteine-type endopeptidase activity, regulation of peptidase activity, regulation of endopeptidase activity. **D.** Upregulated differential genes were enriched to the top 10 in CD59- set included neutrophil activation involved in immune response, neutrophil activation, neutrophil degranulation, neutrophil mediated immunity, type I interferon signaling pathway, cellular response to type I interferon, response to type I interferon, cellular response to chemical stress, positive regulation of defense response, defense response to virus.
