# Supplementary material for: Identifying Genes Associated With Proliferation, Immunity and Thrombosis in Paroxysmal Nocturnal Haemoglobinuria
Source: J Cell Mol Med. 2024 Dec 13;28(23):e70295. doi: 10.1111/jcmm.70295 (PMC11640899; doi:10.1111/jcmm.70295)
Supplement: Supplementary file 4 — FIGURE S4. (A) Gene correlation analysis via the STRING protein interaction network shows a significant correlation between SELP, vWF, FLT1 and NRP1. (B) Sanger sequencing results of selected genes. SELP (rs116959152), SELP (rs141287418), NRP1 (rs200381308), FLT1 (rs2296191), vWF (rs139196998), SLC15A4 (rs536892230), SLC15A4 (rs200862556) and ABCA13 (rs148629439) were identified (details are shown in Table S6). [file JCMM-28-e70295-s004.docx]

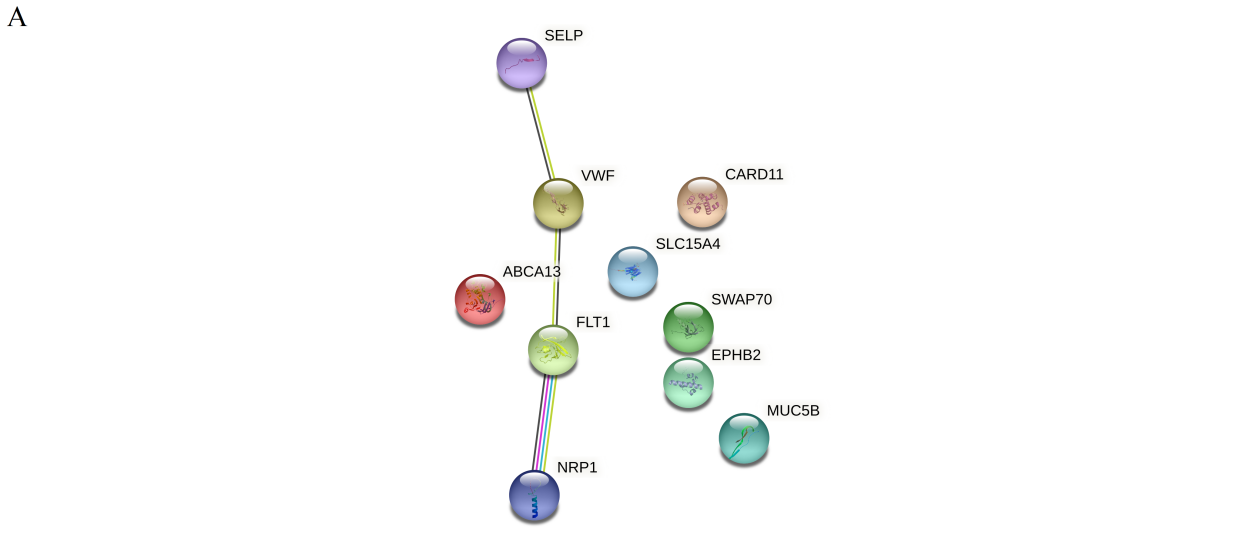


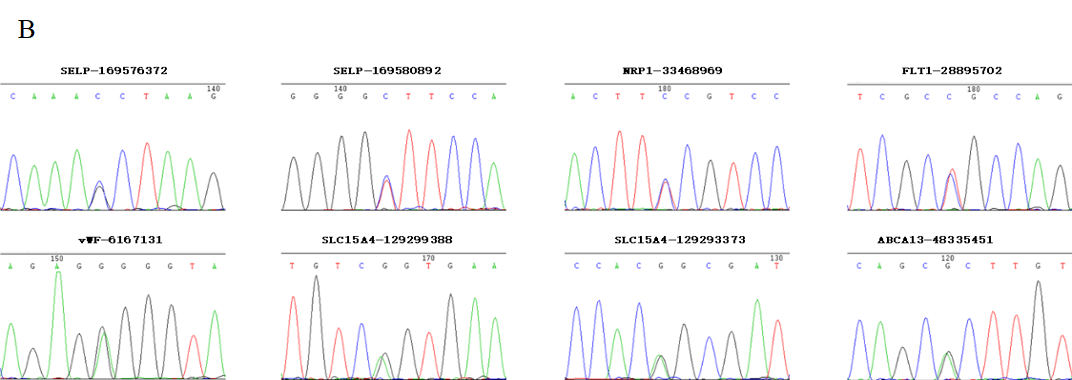


Supplementary Figure 4. A. Gene correlation analysis via the STRING protein interaction network showed a significant correlation between SELP, vWF, FLT1 and NRP1. B. Sanger sequencing results of selected genes. SELP(rs116959152), SELP(rs141287418), NRP1(rs200381308), FLT1(rs2296191), vWF(rs139196998), SLC15A4(rs536892230), SLC15A4(rs200862556) and ABCA13(rs148629439) were identified(details were showed in supplementary table 6).
