# Supplementary material for: Identifying Genes Associated With Proliferation, Immunity and Thrombosis in Paroxysmal Nocturnal Haemoglobinuria
Source: J Cell Mol Med. 2024 Dec 13;28(23):e70295. doi: 10.1111/jcmm.70295 (PMC11640899; doi:10.1111/jcmm.70295)
Supplement: Supplementary file 5 — FIGURE S5. (A) The expression of vWF in CD59+ cells, CD59‐ cells and healthy control cells was analysed by western blotting. (B) Band ‘a’ indicates a significant difference in protein expression between healthy controls and PNH patients by SDS–PAGE with Coomassie brilliant blue staining under the same loading conditions. [file JCMM-28-e70295-s012.docx]

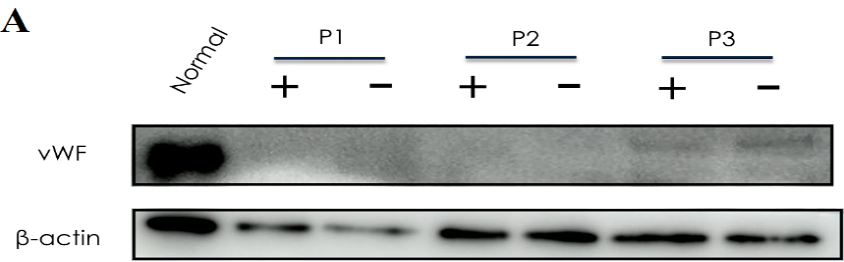


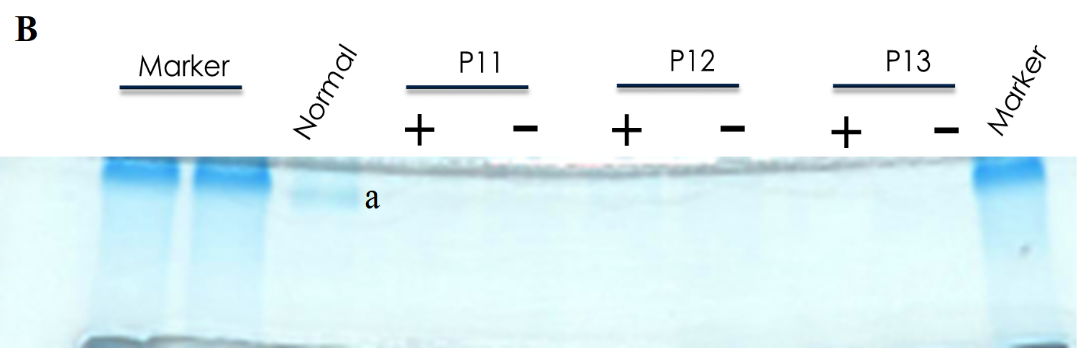


Supplementary Figure 5.A. The expression of vWF in CD59+ cells, CD59- cells and healthy control cells was analysed by western blotting. B. Band ‘a’ indicates a significant difference in protein expression between healthy controls and PNH patients by SDS‒PAGE with Coomassie brilliant blue staining under the same loading conditions.
