# Supplementary material for: Identifying Genes Associated With Proliferation, Immunity and Thrombosis in Paroxysmal Nocturnal Haemoglobinuria
Source: J Cell Mol Med. 2024 Dec 13;28(23):e70295. doi: 10.1111/jcmm.70295 (PMC11640899; doi:10.1111/jcmm.70295)
Supplement: Supplementary file 6 — TABLE S1. Primers for Sanger sequencing. [file JCMM-28-e70295-s013.docx]

Supplementary Table 1.Primers for Sanger sequencing

| **Gene** | **Position** | **Primer** |
| --- | --- | --- |
| CARD11 | 2985361 | F-GCAAGGCAAGGACAAGATCTAC |
|  |  | R-CTATGTGGTCTTCTTGGAGAGC |
| SELP | 169576372 | F-AGAATTCCAGTTTCCAGTAGCC |
|  |  | R-ATAAAATCCTTCATGTGCCG |
|  | 169580892 | F-AAATCCTACCCTCACAGGTTG |
|  |  | R-CTGGACAAACATCATTAGGGTC |
| EPHB2 | 23238949 | F-CCGCAATCATGCAGAAATACTC |
|  |  | R-AGAAGTGGCTGGGCACTTACTC |
|  | 23240387 | F-AAAGGGACATTCTCCGGGTT |
|  |  | R-GGCTGTGCGCGTGATATGTA |
| ABCA13 | 48314102 | F-GGCTTCATTTAATAACACTGGG |
|  |  | R-GGGTACGCATGACAGAAGTTAC |
|  | 48335451 | F-ATTGAAAAGATATGGTCCTCGC |
|  |  | R-TGCTGCTGTTACATTTTACCAC |
|  | 48414007 | F-TAAATGACTGCCTGAAGCGATG |
|  |  | R-TGATTGTTCTCAAAACTGGTGC |
|  | 48520734 | F-GTGGTATTCTGGCATAATTCTC |
|  |  | R-TGAAAGTCAGCAGTAAGGAATC |
| NRP1 | 33468969 | F-CAACACCAGCATCTGATTATTC |
|  |  | R-TACTGTGCCTGTTGGCATAATG |
|  | 33515188 | F-GCCAGACAGAAAGCTACCAAAC |
|  |  | R-GAATGAGGCCCTGTTGATCC |
|  | 33559615 | F-CCTGAGTAGCTGGGATTACTGG |
|  |  | R-TTTCTTCCTGGCACACTATGC |
| MUC5B | 1256409 | F-CTGTGAGCAGGCACCATTG |
|  |  | R-TCACCTCCACGAAGAGCTTG |
|  | 1280193 | F-GTGCATCTATGCTCCATCTGAG |
|  |  | R-AGGGAGCCAGGATAAGTGACTC |
|  | 1283135 | F-GCCACTGCTGTCTGAGAACG |
|  |  | R-ACCTCAGCCCTGAGTTACCC |
| SWAP70 | 9771575 | F-ACCTGATCACTAACTGGGGAC |
|  |  | R-TCAGGTAACCCTTGTTTGCAGG |
| SLC15A4 | 129293373 | F-GACATGCACTTCCTGAACAACC |
|  |  | R-TAAACGCCTCCTCCTGTGTTTC |
|  | 129299388 | F-GGAGAGAAGACATGTAAACCAC |
|  |  | R-TTATTGGAGCATTAACCTGG |
| VWF | 6092312 | F-CGTGCAGGTGTGACCCTAAC |
|  |  | R-GAATGTAGCATCCCACTCACAG |
|  | 6153591 | F-CTGCCTACAAGAAAACTGAAGG |
|  |  | R-GTGGGCAACTCTGAGTCTCTTG |
|  | 6167131 | F-CTGCACTAATGTGGAGACCTCG |
|  |  | R-GCTAAACAACTATGCCGCTGC |
| FLT1 | 28895702 | F-TGATGGTTTTCAGGGACTACAG |
|  |  | R-CCCTTCACAGCATGTGAAATG |
|  | 29005398 | F-TTGGCAACGCTGAACTATGC |
|  |  | R-TACGCTCTAGGTGGATTTGAC |
|  | 29041087 | F-GGAATCTCTTTCCTAACCCG |
|  |  | R-AAATGGTCTTTGCCTGAAATGG |
