# Supplementary material for: Identifying Genes Associated With Proliferation, Immunity and Thrombosis in Paroxysmal Nocturnal Haemoglobinuria
Source: J Cell Mol Med. 2024 Dec 13;28(23):e70295. doi: 10.1111/jcmm.70295 (PMC11640899; doi:10.1111/jcmm.70295)
Supplement: Supplementary file 7 — TABLE S2. Primers for qPCR. [file JCMM-28-e70295-s009.docx]

Supplementary Table 2. Primers for qPCR

| **Gene** | **Primer** |
| --- | --- |
| β-actin | F-TAGTTGCGTTACACCCTTTCTTG |
|  | R-TCACCTTCACCGTTCCAGTTT |
| NRP1 | F-TGGAATGTTGGGTATGGTGTC |
|  | R-AGGAATGAGGTGCGGGTG |
| SELP | F-AGCATGGACTTATCATTACAGCAC |
|  | R-TTCATTATCAGCCCAGTTCTCA |
| FLT1 | F-CAAATGACCTGGAGTTACCCTG |
|  | R-ATGATGGTCCACTCCTTACACG |
| vWF | F-CTCCGCATCCAGCATACAGT |
|  | R-AAGTCGTCGCCCTGGTTG |
