# Supplementary material for: Identifying Genes Associated With Proliferation, Immunity and Thrombosis in Paroxysmal Nocturnal Haemoglobinuria
Source: J Cell Mol Med. 2024 Dec 13;28(23):e70295. doi: 10.1111/jcmm.70295 (PMC11640899; doi:10.1111/jcmm.70295)
Supplement: Supplementary file 8 — TABLE S3. Clinical characteristics of the different cohorts. [file JCMM-28-e70295-s007.docx]

Supplementary Table 3. Clinical characteristics of the different cohorts

|  | Total(N=86) | Sample sorted by microbeads(N=6)* | Sample sorted by flow cytometry(N=7)* | Sample without sorting(N=73)* | P value |
| --- | --- | --- | --- | --- | --- |
| Sex(M/F) | 53/33 | 5/1 | 5/2 | 43/30 | 0.425 |
| Age at diagnosis | 34.5(11,75) | 34(27,58) | 32(19,51) | 35(11,75) | 0.748 |
| Diagnosis BMF-PNH/Classic PNH | 44/42 | 2/4 | 2/5 | 40/33 | 0.603 |
| Thrombosis (with/without) | 29/57 | 4/2 | 2/5 | 23/50 | 0.215 |
| PNH clone (%) | 71.98±31.32 | 83.7±14.2 | 85.1±11.3 | 69.9±33.1 | 0.615 |
| RET# (×10^9^/L) | 161.6±107.9 | 135.9±40.0 | 190.5±131.7 | 160.4±109.4 | 0.7 |
| WBC(×10^9^/L) | 4.52±2.36 | 4.6±1.6 | 4.3±1.4 | 4.5±2.5 | 0.777 |
| Neut#(×10^9^/L) | 2.3±1.9 | 1.0±0.7 | 2.1±1.2 | 2.4±2.0 | 0.35 |
| HGB(g/L) | 80.15±22.2 | 82±34.3 | 78.1±18.1 | 80.2±21.8 | 0.947 |
| PLT(×10^9^/L) | 136.9±89.19 | 126.5±82.6 | 97.6±58.9 | 141.2±91.7 | 0.544 |
| LDH(U/L) | 1348.75±853.65 | 1465.2±997.1 | 1710.3±872.9 | 1306.3±844.2 | 0.454 |
| Tbil(mmol/L) | 25.49±14.72 | 33±19.4 | 34±13.6 | 24.1±14.2 | 0.095 |
| Dbil(mmol/L) | 7.27±3.46 | 8.9±5.6 | 10±3.4 | 6.9±3.2 | 0.062 |
| Scr(μmol/L) | 70.46±21.91 | 77.5±29.8 | 66.7±24 | 70.3±21.3 | 0.726 |
| SF (ng/mL) | 553.2±1497.6 | 253.7±256.0 | 273.5±471.6 | 553.2±1497.6 | 0.86 |

Abbreviations: DBil: direct bilirubin; HGB: haemoglobin; LDH: lactate dehydrogenase; Neut#: neutrophil count; PLT: platelet count; Ret: reticulocyte count; Scr: serum creatinine; SF: serum ferritin; TBil: total bilirubin; WBC: white blood cell. * P value indicates comparisons among cohorts 1, 2 and 3.

* Eighty-six PNH patients were included; median age was 42 (range 21-68) years-old, and there were 53 males and 33 females (Table 3 in Supplementary Data 2). Six patients who underwent cell sorting for WES (cohort 1), 7 patients who underwent cell sorting for mRNA and protein expression verification (cohort 2), and the remaining 73 patients (cohort 3). There was no difference in baseline clinical features among the different cohorts (P>0.05). All of the patients had not received component inhibitors.
