# Supplementary material for: Identifying Genes Associated With Proliferation, Immunity and Thrombosis in Paroxysmal Nocturnal Haemoglobinuria
Source: J Cell Mol Med. 2024 Dec 13;28(23):e70295. doi: 10.1111/jcmm.70295 (PMC11640899; doi:10.1111/jcmm.70295)
Supplement: Supplementary file 9 — TABLE S4. Clinical characteristics of patients treated with immunosuppressive therapy (IST). [file JCMM-28-e70295-s008.docx]

Supplementary Table 4. Clinical characteristics of patients treated with immunosuppressive therapy (IST)

|  | Response to IST(N=24) | Without response to IST(N=14) | P value |
| --- | --- | --- | --- |
| Sex(M/F) | 12/12 | 6/8 | 0.75 |
| Diagnosis | 22/2 | 12/2 | 0.72 |
| Age at diagnosis | 33(11,69) | 22.5(12,68) | 0.22 |
| PNH clone (%) | 68.5(4,99) | 91.2(7,99) | 0.10 |
| RET(%) | 4.11(1.02,10.01) | 6.75(1.77,14.04) | 0.04 |
| RET# (×10^9^/L) | 86.94(12.138,268.268) | 160.304(49.72,415.584) | 0.10 |
| WBC(×10^9^/L) | 3.48(1.71,7.46) | 3.52(2.61,12.4) | 0.60 |
| Neut#(×10^9^/L) | 1.785(0.18,5.31) | 1.805(0.84,6.6) | 0.30 |
| PLT(×10^9^/L) | 78.5(15,296) | 68.5(23,219) | 0.93 |
| LDH(U/L) | 797.5(183,2744) | 1559(288,2660) | 0.01 |
| Tbil(mmol/L) | 17.7(4.9,41.3) | 26.7(7.5,44.1) | 0.09 |
| Dbil(mmol/L) | 5.5(1.9,17.6) | 7.15(3.2,11.3) | 0.41 |
| Scr(μmol/L) | 76(39,160) | 61.5(37,121) | 0.08 |
| SF (ng/mL) | 474.5(15,3785) | 173(13,5311) | 0.55 |

Abbreviations: DBil: direct bilirubin; HGB: haemoglobin; LDH: lactate dehydrogenase; Neut#: neutrophil count; PLT: platelet count; Ret: reticulocyte count; Scr: serum creatinine; SF: serum ferritin; TBil: total bilirubin; WBC: white blood cell. * P value indicates comparisons between patients response to IST or without response to IST.
