# Supplementary material for: Identifying Genes Associated With Proliferation, Immunity and Thrombosis in Paroxysmal Nocturnal Haemoglobinuria
Source: J Cell Mol Med. 2024 Dec 13;28(23):e70295. doi: 10.1111/jcmm.70295 (PMC11640899; doi:10.1111/jcmm.70295)
Supplement: Supplementary file 10 — TABLE S5. Results of mass spectrometry. [file JCMM-28-e70295-s002.docx]

Supplementary Table 5. Results of mass spectrometry

| **Sample** | **Protein name** | **mol. wt** | **calc pI**  **value** | **seq**  **coverage** | **mascot**  **score*** | **Gene name** | **Function** |
| --- | --- | --- | --- | --- | --- | --- | --- |
| Control | Histamine N-methyltransferase | 33274 | 5.18 | 58 | 44 | HNMT | Participates in histamine metabolism and inactivates histamine through n-methylation and plays an important role in degrading histamine and regulating airway response to histamine. |
| P11-59+ | Glycylpeptide N-tetradecanoyltransferase 2 | 56944 | 7.23 | 34 | 51 | NMT2 | Participates in regulating the function and localization of signaling proteins by adding myrisyl groups to n-terminal glycine residues of certain cellular and viral proteins. |
| P11-59- | Protein INCA1 | 26800 | 6.92 | 69 | 42 | INCA1 | Participates in negative regulation of cell proliferation and positive regulation of apoptosis, and may play a role in cell cycle control. |
| P12-59+ | Rho guanine nucleotide exchange factor 10 | 151516 | 5.46 | 45 | 41 | ARHGEF10 | Participates in regulation of small GTPase mediated signal transduction and may play a role in myelin development of peripheral nerves. |
| P12-59- | RING finger protein 141 | 25518 | 5.07 | 55 | 41 | RNF141 | Participates in protein autoubiquitination and is the motifs of protein-DNA and protein-protein interactions. |
| P13-59+ | Protein BTG1 | 19197 | 8.35 | 38 | 41 | BTG1 | Antiproliferative protein, a member of the family of antiproliferative genes that regulate cell growth and differentiation. |
| P13-59- | Puromycin-sensitive aminopeptidase | 103211 | 5.49 | 30 | 42 | NPEPPS | Participates in protein autoubiquitination and is the motifs of protein-DNA and protein-protein interactions |

P11,P12,P13: Samples from 3 different PNH patients.

CD59-: CD 59 negative cells.

CD59+:CD59 positive cells.
