# Supplementary material for: Identifying Genes Associated With Proliferation, Immunity and Thrombosis in Paroxysmal Nocturnal Haemoglobinuria
Source: J Cell Mol Med. 2024 Dec 13;28(23):e70295. doi: 10.1111/jcmm.70295 (PMC11640899; doi:10.1111/jcmm.70295)
Supplement: Supplementary file 12 — Data S1. [file JCMM-28-e70295-s010.docx]

**PCR amplification reaction system**：10×buffer 2.5ul,Mg^2+^ 2mM,dNTP 0.5ul(10mMOL/mL),Taq polymerase 0.5ul(5U/ul),template ~20ng,Primers 1ul(10uM),ddH2O was used to supplement the total system to 25ul

2.PCRtouch down amplification reaction procedure:

95℃ 5min

95℃  30S

65℃-50℃ 30S 30cyc(Drop 0.5 ℃ per cycle)

72℃  60S

95℃  30S

50℃  30S 10cyc

72℃ 60S

72℃  8min

4℃ storage until use
