# Supplementary material for: Identifying Genes Associated With Proliferation, Immunity and Thrombosis in Paroxysmal Nocturnal Haemoglobinuria
Source: J Cell Mol Med. 2024 Dec 13;28(23):e70295. doi: 10.1111/jcmm.70295 (PMC11640899; doi:10.1111/jcmm.70295)
Supplement: Supplementary file 13 — Data S2. [file JCMM-28-e70295-s005.docx]

**First screened target genes in detail as follows:**

1.Eighteen genes up-regulated in CD59+ but down-regulated in CD59: four genes in immunty, namely FOXP3, RORC, RORA, LY9. Fourteen genes in proliferation/apoptosis, namely CD81, ADA, CD320, TNFRSF4, CD40LG, CARD11, BCL2, CD6, IL2RA, FOXP3, TMIGD2, CD3E, SLC7A1, CD28. No genes related to thrombus was enriched.

2.One hundred and seventy-six genes down-regulated in CD59+ but up-regulated in CD59-:one hundred and four genes in immunity, namely ADGRE2, AGPAT2, ANPEP, ANXA3, APAF1, APOA2, ARHGAP9, ARRB2, ATG7, ATP8B4, AZU1, BCL6, BST1, C1QA, C1QB, C1QC, CAP1, CCL2, CD300LF, CD63, CDA, CEACAM3, CEBPB, CFP, CKAP4, CLEC4A, CLEC4D, CLEC5A, CLEC6A, CLEC7A, CREG1, CTSA, CTSZ, CYSTM1, DDX3X, EREG, FCAR, FCER1G, FCN1, FFAR2, FOLR3, FTH1, FTL, FUT7, FZD5, GAB2, GPX1, HCK, HK3, HLX, HP, ICAM1, ICAM3, IL18, IL1B, IL1R1, IL27, IRF7, ITGAM, ITGAX, JAK2, KIR2DL1, LILRA2, LILRB1, LILRB2, LILRB3, LILRB4, LOXL3, LST1, MCEMP1, MERTK, MGAM, MGST1, MME, NECTIN2, NLRP6, NOD2, P2RX1, PAK1, PARP9, PGLYRP1, PLAUR, PLSCR1, PRAM1, PTX3, PVR, PYGL, QPCT, RNASE3, RNASET2, ROCK1, S100A12, S100A8, S100P, SERPINA1, SIGLEC5, SIRPB1, SLCO4C1, STX7, TCN1, TRIB1, TRPM2, TYROBP, VNN1. Nineteen thrombus related genes, namely ANXA5, ARRB2, CLEC1B, DGKG, ENTPD1, EPHB2, F5, FCER1G, JAK2, MERTK, NFE2, P2RX1, PLAUR, PLEK, PLSCR1, SERPINA1, SERPINB2, SLC7A11, ST3GAL4. Fifty-three proliferation/apoptosis related genes, namely APAF1, ARRB2, ATP2A1, BCL2A1, CCL2, CD300LF, CEBPB, CFLAR, CLEC7A, CPEB4, CTSL, DAPK2, DDIAS, DDX3X, EREG, FCN1, FNIP1, FOXO3, GBE1, GPX1, HCAR2, HIF1A, HIP1, ICAM1, IFI6, IL18, IL1B, IRF7, ITGAM, ITPRIP, JAK2, JMJD6, KLF4, LRRK2, MCL1, MERTK, NAIP, NLRP12, NMNAT1, NOD2, NQO2, P2RX1, PAK1, PLAUR, PPIF, RB1CC1, S100A8, SLC7A11, SNCA, STK3, TYROBP, VEGFA, VNN1.

3.Ninety-four genes down-regulated in ‘Dirty’ set that related to immunity, namely ADGRE5, ALOX15, BANK1, BLK, BLNK, C5AR1, CASP3, CCR2, CCR3, CD180, CD19, CD22, CD36, CD40, CD79A, CD79B, CHI3L1, CLEC4E, CLU, CMKLR1, CNR2, COPB1, CPPED1, CR1, CR2, CRISPLD2, CX3CR1, CXCL5, CXCR2, DOK3, ENPP3, FCER2, FCGR2A, FCGR2B, FLT3, FPR1, GBP1, GCA, HBB, HLA-DOB, HLA-DPA1, HLA-DQB1, HSPA6, IDO1, IFI16, IFIT1, IFNGR1, IGLL5, IL13RA1, IL18RAP, IL1RAP, IL5RA, IL6R, IRF4, KIR3DL1, KLRD1, KLRF1, LRG1, MNDA, MS4A1, MS4A3, NCR1, PAX5, PECAM1, PF4, PPM1B, PRF1, PROS1, PTK2, RNASE6, SEC14L1, SELL, SEMA7A, SERPING1, SH2B2, SH2D1B, SIRPA, SLC11A1, SLC15A4, SLC27A2, SLC2A5, SLC44A2, SWAP70, SYNGR1, TIMP2, TLR2, TLR4, TLR8, TMEM176B, TRIM21, TXNDC5, VPREB3, XCL1, XRCC4. Thirty-three genes down-regulated in‘Dirty’set that related thrombus, namely ADRA2A, BLK, CAPZA1, CD36, CD40, CD9, CLU, CTSW, EGF, F11R, F13A1, GP9, HBB, HPS6, ITGA2B, ITGB3, ITPK1, MPIG6B, P2RX5, PEAR1, PECAM1, PF4, PRKAR2B, PROS1, SELP, SERPING1, SH2B2, SPARC, TAGLN2, TBXA2R, TLR4, TREML1, VWF. Fourty-eight genes down-regulated in ‘Dirty’ set that related to proliferation/apoptosis, namely ALOX15, ATOH8, BLK, BMP6, C5AR1, CASP3, CCR2, CCR3, CD151, CD180, CD19, CD22, CD36, CD40, CD79A, CLU, CR1, CR2, EGF, ENPP3, FCGR2B, FGFR2, FLT1, FLT3, HLA-DPA1, IDO1, IL5RA, ITGB3, LAMC1, LRG1, MNDA, MS4A1, MTSS1, NRP1, PEAR1, PROK2, PTEN, PTK2, RHOBTB1, SLC11A1, SPARC, TEK, TGFA, TIE1, TLR4, TXNDC5, VASH1, XCL1.

4.Thirty-two genes up-regulated in ‘Dirty’ set that related to immunity, namely ABCA13, A2M, BPI, CCL5, CD177, CD83, CEACAM6, CHIT1, CXCL1, CXCL8, DUSP10, HSP90AA1, KRT6A, LAG3, LCN2, LTA, LTF, MATR3, MMP8, MMP9, MS4A3, MUC1, MUC5B, NLRC5, OLFM4, OLR1, ORM1, PLAU, RETN, RGCC, RPL30, USP18. Neither genes related to proliferation/apoptosis nor thrombus was enriched.
